# Flexural Behavior of Ultra-High-Performance Fiber-Reinforced Concrete Beams after Exposure to High Temperatures

**DOI:** 10.3390/ma14185400

**Published:** 2021-09-18

**Authors:** How-Ji Chen, Chien-Chuan Chen, Hung-Shan Lin, Shu-Ken Lin, Chao-Wei Tang

**Affiliations:** 1 Department of Civil Engineering, National Chung-Hsing University, No. 250, Kuo Kuang Rd., Taichung 40227, Taiwan; hojichen@dragon.nchu.edu.tw (H.-J.C.); g2125ster@gmail.com (C.-C.C.); z0813zzz@outlook.com (H.-S.L.); sklin@nchu.edu.tw (S.-K.L.); 2 Department of Civil Engineering and Geomatics, Cheng Shiu University, No. 840, Chengching Rd., Niaosong District, Kaohsiung 83347, Taiwan; 3 Center for Environmental Toxin and Emerging-Contaminant Research, Cheng Shiu University, No. 840, Chengching Rd., Niaosong District, Kaohsiung 83347, Taiwan; 4 Super Micro Mass Research and Technology Center, Cheng Shiu University, No. 840, Chengching Rd., Niaosong District, Kaohsiung 83347, Taiwan

**Keywords:** ultra-high-performance concrete, flexural behavior, high temperature, ductility

## Abstract

Due to the dense structure of ultra-high-performance concrete (UHPC), it is prone to explosive spalling at high temperatures. In this paper, flexural testing of UHPC and high-strength concrete (HSC) beams was carried out at room temperature and after being subjected to different levels of thermal exposure (300–500 °C). The cross-section of the beam specimen was 150 (width) × 200 (depth) mm, and its length was 1500 mm. The flexural and shear design of the beam specimens were carried out in accordance with the ACI 318M-14 code. All of the beams were singly reinforced with two #4 rebars (minimum reinforcement ratio) as a longitudinal tensile reinforcement at the bottom of the specimen and at an effective depth of 165 mm. The flexural load was applied using the three-point load method. The results show that, at room temperature and after being subjected to different thermal exposures, compared with the HSC specimens, the stiffness of the UHPC specimens in the post-cracking stage was relatively larger and the deflection under a given load was smaller. Moreover, whether at room temperature or after exposure to different thermal exposures, the ductility of the UHPC specimens was better than that of the HSC specimens.

## 1. Introduction

Concrete is a cement-based composite material that uses cement combined with aggregates, additives, etc., to form a hydraulic cementitious material [1]. Since the 1970s, due to the successful development of chemical admixtures (especially superplasticizers) and the application of mineral admixtures (mainly ground granulated blast furnace slag, silica fume, fly ash, etc.), various special concrete products have been developed one after another [2,3,4]. Familiar products include, for example, high performance concrete (HPC) and ultra-high-performance concrete (UHPC).

The American Institute of Concrete (ACI Committee 239) defines UHPC as “concrete that has a minimum specified compressive strength of 150 MPa and meets specific durability, tensile ductility, and toughness requirements” [5]. Many researchers point out [6,7,8] that there are three key factors in the production of UHPC, including improved micro and macro properties of the matrix composition, maximum particle packing density, and minimum size defects. The typical composition of UHPC generally includes cement, fine aggregates, fiber, mineral additives, and superplasticizer. The mix proportions of UHPC have several characteristics, including a low water-to-binder ratio, a large amount of very fine powders by using only fine sand for aggregates, a low water content (adding high-dose superplasticizers), and the use of fibers [9,10,11]. UHPC is generally prepared by mixing its constituent materials with water, before setting and hardening it to form concrete with an ultra-high compressive strength, high tensile toughness, and excellent durability. At present, UHPC is applied in major engineering projects such as roads, bridges, and water conservancy projects [12,13,14].

In view of the very broad prospects of UHPC, many researchers are actively engaged in research on its performance, which is of great significance to the development of civil engineering materials. The mechanical properties of UHPC depend on the constituent materials, pouring methods, and curing conditions used. Among them, fiber is the key factor, and the mixing of different types of fibers will cause the performance of the UHPC to change according to the performance of the incorporated fiber. Biswas et al. [15] conducted a detailed literature review on the influence of steel fiber percentage and aspect ratio on the fresh and mechanical properties of UHPC in order to understand the effect of steel fiber on UHPC performance. They found that, due to interlocking between the fibers, an increase in the amount of steel fiber and its aspect ratio had a negative impact on workability. He et al. [16] used glass fiber (GF) and high-performance polypropylene (HPP) fibers to prepare UHPC and studied the effect of these fibers on its mechanical properties. Their research results show that the improvement effect of glass fiber was more significant compared with HPP-UHPC. The compressive, tensile, and flexural strengths of GF-UHPC were increased by approximately 20%, 30%, and 40%, respectively. However, the flexural toughness index of HPP-UHPC was better than that of GF-UHPC.

The influence of the longitudinal reinforcement ratio on the flexural performance of UHPC beams has also aroused great interest among scholars. Kahanji et al. [17] conducted a series of flexural tests on UHPC beams. They found that the bending moment capacity and stiffness increased with increasing fiber volume fraction. You et al. [18] produced UHPC beams with different reinforcement ratios (0–1.71%) to explore their flexural behavior. Their research shows that as the reinforcement ratio increased, the stiffness and load-bearing capacity of UHPC beams after cracking increased, while the cracking load decreased. Hasgul et al. [19] investigated the flexural behavior of UHPC beams with low and high reinforcement ratios. They found that even at high reinforcement ratios, due to the high compressive strength and deformability of UHPC beams, a ductile flexural behavior could still be achieved. On the other hand, Chalioris et al. [20] studied the cyclic response of steel fiber concrete (SFRC) beams under reverse loading. They found that compared with the control reinforced concrete beams, the SFRC beams not only exhibited improved overall hysteresis response and increased energy absorption capacity, but also enhanced cracking modes and changes in failure characteristics from concrete crushing to toughness. In addition, some researchers have explored the influence of steel fibers on the seismic resistance of reinforced concrete structural members. K. Kytinou et al. [21] investigated the hysteresis response of slender and deep SFRC beams under reverse cyclic loading. They found that the hysteresis response of SFRC beams was improved, including residual stiffness, bearing capacity, deformability, energy dissipation capacity, and crack resistance. Furthermore, the freezing and thawing durability of UHPC has also attracted the attention of academic circles. Feo et al. [22] conducted a series of flexural tests on high-performance fiber reinforced concrete (HPFRC) prismatic specimens to evaluate the influence of fiber content and the number of freeze-thaw cycles on the flexural behavior of the specimens. Their research shows that the bending response of the HPFRC specimens was greatly affected by fibers. Compared with ordinary concrete specimens, the HPFRC specimens had good energy absorption capacity, and thus exhibited better initial crack strength and post-crack behavior.

Fire has always been a serious threat to building structures, and there is no exception for building structures made with UHPC. The question of how to ensure that UHPC has good fire resistance is one of the hot topics of current academic research. Because UHPC has a relatively dense microstructure, its performance changes under the influence of fire and high temperatures; results of these performance changes, such as mechanical degradation and explosive spalling, have attracted widespread attention from scholars and research related to them has frequently been reported. In a high temperature environment, UHPC is more prone to explosive spalling than ordinary concrete, due mainly to the compactness of its structure. Therefore, many scholars propose that the use of polypropylene (PP) fiber may avoid this phenomenon [23,24,25]. This is attributable to the fact that polypropylene fibers melt at high temperatures to form channels, which can release accumulated water vapor pressure. According to Schneider and Horvath [26], the most common PP fiber type begins to melt at about 160 °C, the fiber disintegrates at about 205 °C, and the degradation reaction is completed at about 380 °C. However, Poon et al. [27] show that once the temperature of the specimen exceeds 600 °C, the compressive strength of UHPC blended with polypropylene fiber decreases sharply, indicating that polypropylene fiber has lost its positive effect. Tai et al. [28] studied the residual mechanical properties of UHPC after high temperature. They found that the residual compressive strength of UHPC continued to increase after exposure to temperatures from 200 to 300 °C; however, as the temperature continued to rise and exceeded 300 °C, the residual compressive strength of UHPC began to decrease. Chen et al. [29] explored the mechanical properties of UHPC at room temperature and after exposure to high temperatures. Their results show that the residual mechanical properties (such as compressive strength, flexural strength, and splitting strength) of UHPC at 300, 400, and 500 °C did not decrease significantly. But when the temperature was 600 °C, UHPC spalled explosively, and its residual mechanical strength obviously declined. Liang et al. [30] studied the coupling effect of temperature and impact load on the tensile strength of fiber-added UHPC. They found that once the temperature exceeded 400 °C, the splitting tensile strength of the UHPFC declined significantly, but it still maintained 41% of its room temperature strength at 800 °C. In addition, the combined effects of temperature and impact load had different effects on the splitting tensile strength and compressive strength of the UHPFC.

Lu et al. [31] studied the influence of typical auxiliary cementitious materials (SCM) on the thermal properties and microstructure of HPC. Their results reveal the potential of using SCM in combination to improve the high-temperature performance of HPC. In addition, the combined use of SCMs could delay and reduce the micro-cracking of high-performance concrete at high temperatures. Smarzewski [32] explored the toughness development of UHPC after exposure to different thermal exposures. They conducted flexural toughness tests on two types of concrete, one ordinary UHPC and the other UHPC with different types of fibers, the volume fractions of which were 0.5%, 1%, 1.5%, and 2%. They found that, in some cases, the load–deflection curve of fiber-added UHPC exhibited a double-peak response. They pointed out that the first peak was related to the characteristics of UHPC, while the second peak was related to the bridging characteristics of the fiber. In addition, under bending load, the toughness of UHPC declined with increasing temperature. Chen et al. [33] studied the mechanical properties of cement-based composite systems repaired by UHPC after exposure to high temperatures. Their results indicated that as the temperature increased, the residual mechanical properties of the system first increased and then decreased. Once the temperature reached 500 °C, the residual compressive strength, bonding strength, and flexural strength of the UHPC-repaired cementitious composite system decreased by about 20%, 30%, and 15%, respectively.

Kahanji et al. [34] studied the spalling phenomenon of UHPC beams at high temperatures. The test parameters included steel fiber content, type of fiber used (steel fiber and polypropylene fiber), and load level. The fire performance of UHPC beams was tested under three load levels (20%, 40%, and 60% of the ultimate load at room temperature) and when exposed to a standard fire curve (ISO 834). Their test results show that the spalling phenomenon was affected by the load level; under the same loading conditions, the spalling of a beam with a steel fiber content of 4% was lower than that of a beam with a steel fiber content of 2%. Banerjee et al. [35] presented an experimental result of ultra-high-performance fiber concrete (UHPFRC) beams tested under the combined effects of structural load and fire exposure. The main purpose was to test five large UHPFRC beams manufactured with different mix designs to evaluate their structural behavior and spalling phenomena under general environmental and fire conditions. Their test results show that, compared with normal-strength concrete or high-strength concrete beams, UHPFRC beams were prone to explosive spalling in the compression zone (side) of the beam section, resulting in lower fire resistance. When polypropylene fibers were present in UHPFRC, the degree of spalling caused by fire damage could be reduced, resulting in a higher fire resistance.

UHPC has the advantages of light weight, high toughness, high durability, etc., meaning that it can meet the requirements of some special buildings and has wide application prospects. Generally speaking, HSC and HPC have worse fire resistance and high-temperature performance than normal-strength concrete [36]. The compressive strength of UHPC is significantly higher than that of HSC and HPC, and its microstructure is extremely dense. Therefore, many scholars have studied its fire resistance and high-temperature performance. However, few related studies have been carried out on the flexural behavior of UHPC beams after being subjected to different thermal exposure conditions. In view of this, this article aimed to study the flexural behavior of UHPC beams at room temperature and after exposure to different thermal exposures. Under normal temperature conditions, flexural tests were performed on UHPC beam specimens exposed to different target temperatures in a laboratory electric furnace to understand the changes in their peak load and load–deflection characteristics. In addition, different deflection ductility indices were used to characterize the ductility changes of UHPC beam specimens after different thermal exposures.

## 2. Experimental Study

### 2.1. Materials

**Figure 1:**
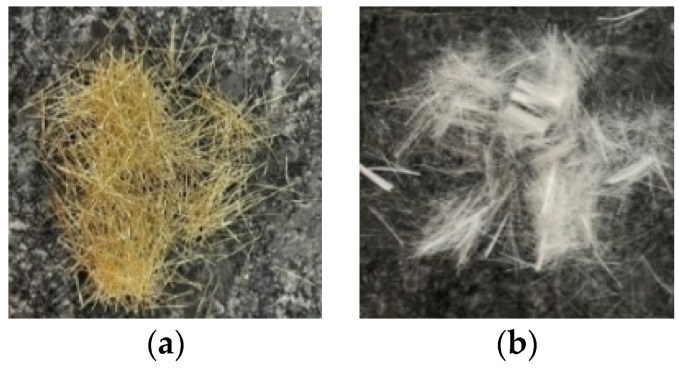
The appearance of the fibers: (**a**) steel fibers and (**b**) polypropylene fibers.

The materials used in this study include water, cement, mineral admixtures (silica fume and ultra-fine silica powder), fine aggregates (quartz sand), chemical admixtures (superplasticizer and viscous agent), fibers, and steel rebars. The properties and sources of these materials are as follows:

- Water: general tap water that met the quality requirements of concrete mixing water.
- Cement: ordinary Portland Type I cement produced by the Taiwan Cement Corporation. Its specific gravity and fineness were 3.15 and 3400 cm^2^/g, respectively.
- Silica fume: Elkem Microsilica 940U purchased from Taiwan Sika Co., Ltd., its specific gravity was 2.1, and its silicon dioxide (SiO_2_) content was 92.4%.
- Ultra-fine silicon powder: purchased from Dawei Stone Industry Company (Jhubei, Taiwan), its specific gravity and silicon dioxide content were 2.73 and 92%, respectively, and the average particle size was 0.075–0.225 μm.
- Fine aggregate: purchased from Jinjing Silica Sand Co., Ltd.; it belonged to quartz sand and contained two different grades, namely Type A and Type B. The properties and composition of these fine aggregates are shown in Table 1, and the particle size distribution is shown in Table 2. Among the fine aggregates, 80% are type A and 20% are type B.
- Superplasticizer: R-550, a product of Taiwan Sika Company that met the requirements of ASTM C494-81 Type F.
- Viscous agent: purchased from Guanghui Building Material Co., Ltd.
- Fiber: purchased from Gulili Co. Ltd., short micro steel fiber (according to ASTM A820) with a length of 13 mm and a diameter of 0.2 mm and polypropylene fiber with a length of 12 mm and a diameter of 0.05 mm, as shown in Figure 1. The basic properties of the two fibers are shown in Table 3.
- Steel rebar: SD280W #3 and #4. Their various physical and mechanical properties are shown in Table 4.

### 2.2. Mix Proportions of Concrete and Casting of Specimens

The mix proportions of the concretes are shown in Table 5. Among them, high-strength concrete (HSC) is the control group, and its mix designation is C1; ultra-high-performance concrete (UHPC) is the experimental group, and its mix designation is E1. The mixing of the concrete mixtures was carried out with an electric mixer. Before mixing, the fine aggregate was processed into an absolutely dry state because its water absorption was quite low. Next, the silica fume, aggregates, and half of the content of steel fiber and polypropylene fiber were placed in the mixing barrel. As the fibers were not easy to disperse, they were evenly dispersed by manual methods and fully dry mixed for a few minutes until they were uniform. After that, the ultra-fine silicon powder, cement, and the other half of the steel fiber and polypropylene fiber were put into the mixing barrel and fully dry mixed again. Then, the water was slowly poured into the mixer. After mixing for about 1 min, the superplasticizer and viscous agent were slowly poured into the mixture and mixed thoroughly to form a homogeneous fresh concrete.

**Figure 2:**
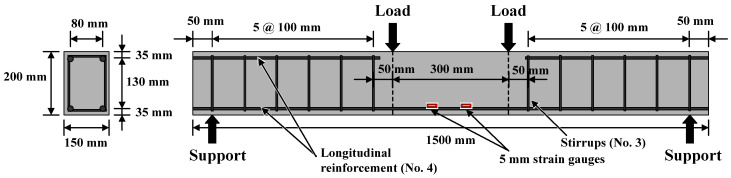
The details of the reinforcement used for the beam specimens.

**Figure 3:**
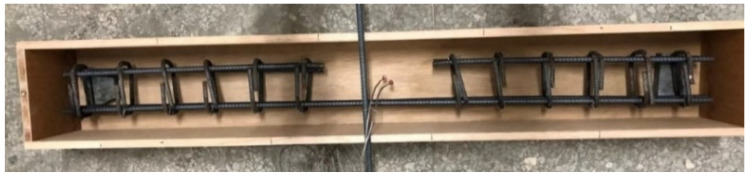
The configuration of the steel rebar and thermocouple.

The flexural and shear designs of all beam specimens were carried out in accordance with the ACI 318M-14 design code. All the beams were singly reinforced with two #4 rebars (minimum reinforcement ratio) as a longitudinal tensile reinforcement at the bottom of the specimen and at an effective depth of 165 mm. In addition, two #4 rebars at the top were used for fixing stirrups (#3 rebar), and they were cut at the mid-span of the specimen. The beam specimen was equipped with a suitable concrete protective layer and the length from the center of the longitudinal steel bar to the surface of the specimen was 35 mm. The specimen was designed to resist shear failure when the longitudinal tensile reinforcement yielded so as to obtain pure flexural failure in the center of the beam span. For this reason, from the beam end support point to one-third of the span of each side, #3 was used as stirrups with a spacing of 100 mm. The size of the beam specimen and the detailed configuration of the steel rebars are shown in Figure 2. In addition, thermocouples were embedded 3.5 and 7 cm from the side surface of the specimen in the midpoint of the beam span, as shown in Figure 3. Before the concrete beam was poured, the thermocouple was fixed at the planned buried position with a temporary steel support. When pouring, the mixture was poured in slowly to avoid damaging the thermocouple. In this study, a four-point loading method was used and a shear span ratio (*a*/*d*) of 3.33 was used.

Once the concrete was mixed to a homogeneous state, the fresh properties of each mixture were immediately measured and recorded. Then, cylindrical specimens of 100 mm diameter × 200 mm height and beam specimens of 150 mm × 200 mm × 1500 mm were cast in accordance with the relevant regulations of ASTM and CNS. For each concrete mixture, 24 cylindrical specimens were cast to carry out the compression test. A wooden mold was prepared to cast the beam specimen, and eight specimens (16 in total) were cast for each concrete mixture. After the wooden mold was assembled, a layer of release agent for concrete was applied to the inside of the mold, a cement block was placed to cushion the thickness of the protective layer, then the steel rebars were placed in the mold. When casting the beam specimens, the concrete was poured in three layers. The amount of pouring needed for each layer was about one third of the beam volume, and internal vibration tamping was applied to each layer for about 30 s. After pouring was completed, we used a trowel to smooth the surface of the beam. The specimen was disassembled after 24 h. The cylindrical specimens were then placed in an indoor curing pool for water curing, were taken out one day before the test age, and subjected to compression tests at room temperature and high temperature. The beam specimens were too large to be cured in the water in the curing pool, so they were cured with indoor air while the daily temperature and humidity changes in the room were recorded. The specimens were not taken out until the day before the test age (56 days) for the flexural test at room temperature and after exposure to high temperatures.

### 2.3. Test Methods and Instrumentation

The testing of the fresh and hardened properties of the UHPC were carried out according to the ASTM specifications listed in Table 6 [37,38,39,40,41]. The test age of the cylindrical specimen was 56 days, and an average of three specimens was taken. After the beam specimen had been cured, flexural tests at room temperature and after exposure to high temperatures were carried out in accordance with the ASTM C1609/C1609M-19a specification [41].

**Figure 4:**
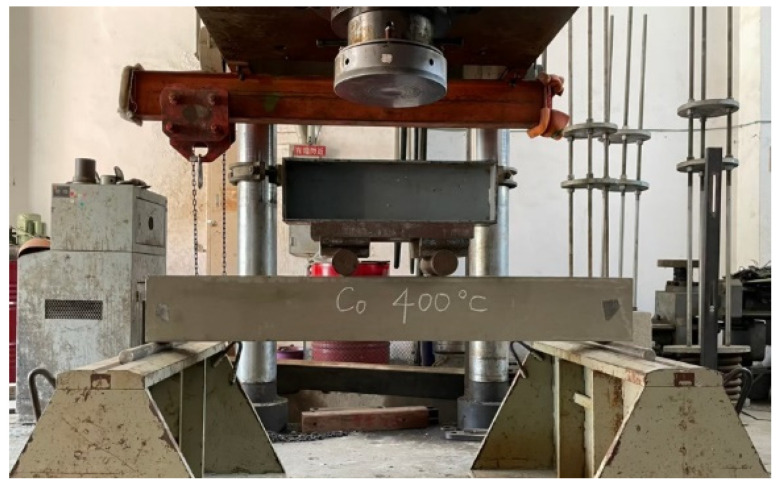
The general flexural test setup for the beam specimens.

At each target temperature, the control group and the experimental group have two beam specimens for flexural tests. The installation of the specimen is shown in Figure 4. An electro-hydraulic servo actuator with a maximum load of 6000 kN was used to apply a monotonic load, and an additional force sensor was installed to ensure the accuracy of the load data. The beam specimen was placed on the two supports of the testing machine and the load was applied using the three-point load method (with the use of the transfer steel beam to create the load position shown in Figure 4; the load was added 15 cm from the center to the left and right). The load was gradually applied at a constant rate of 0.98 kN/s. Each loading time was ten seconds, and the load was maintained at a fixed value for five seconds. The deflection readings at each load step were recorded until the beam specimen failed so as to obtain the relationship curve between the central deflection and the applied load. At each stage of loading, the beam was carefully inspected with a magnifying glass to detect the first crack; then, the flexural load corresponding to the first crack was recorded.

**Figure 5:**
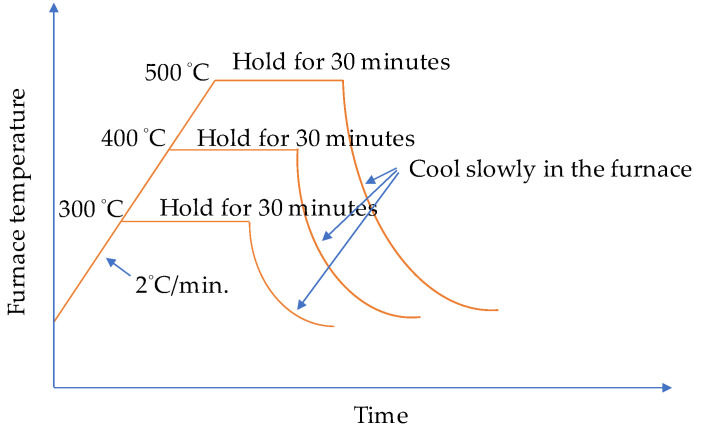
The heating process of the high-temperature test.

Furthermore, there were three types of designed target temperatures: 300, 400, and 500 °C. In order to prevent the beam specimen from spalling during the high-temperature heating, the specimen was pre-dried in a constant temperature furnace at 105 °C before high-temperature heating took place to evaporate part of the moisture inside the specimen. After drying, the specimen was placed in a high-temperature furnace. Only three sides (bottom and two sides) of the specimen were exposed to high temperatures, and the top surface of the beam was insulated with an insulating layer of refractory bricks. This is similar to the actual situation, where there is a concrete slab on the top side of the beam which prevents heat from penetrating from the top. According to literature [29], when high temperature was applied, the heating rate of all samples was set to a fixed value (2 °C/min). Once the target temperature was reached, the holding time was maintained at 0.5 h, so that the entire specimen reached a better thermal stability. Then, the specimen was allowed to cool naturally in the high-temperature furnace to a normal temperature of 23 °C. The schematic diagram of the temperature change in the furnace is shown in Figure 5.

## 3. Experimental Results and Discussion

### 3.1. The Fresh Properties and Compressive Strength of Concrete

The fresh properties and compressive strength test results of the two groups of concrete mixtures are shown in Table 7. It can be seen from Table 7 that the slump of the two groups of concrete specimens reached more than 255 mm, and the slump reached more than 500 mm, indicating that they had excellent flow properties. In addition, Table 7 also lists the unit weight test results of the two groups of concrete. The unit weight of the UHPC was 2316.2 kg/m^3^, while the unit weight of the HSC was 2318.3 kg/m^3^.

**Figure 6:**
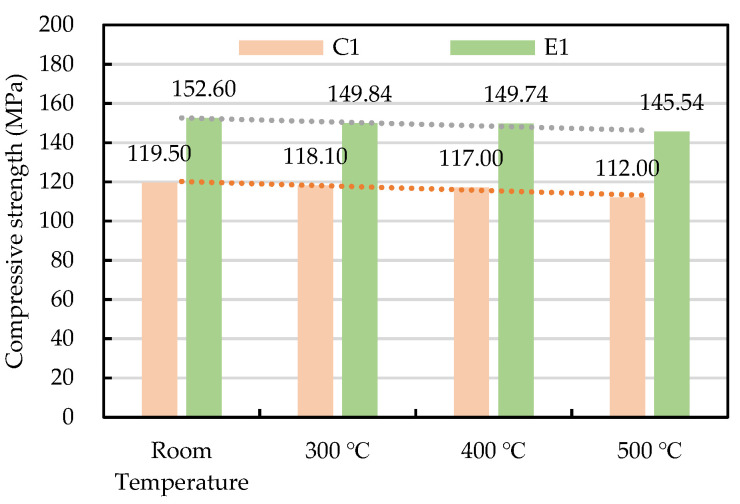
The trend of the residual compressive strength of the concrete after being subjected to high temperatures.

On the other hand, at 56 days of age the HSC and UHPC were tested to determine their compressive strength at room temperature. It can be seen from Table 7 that, due to the low ratio of W/B and the large amount of pozzolanic reaction in the active mineral mixture, the 56 day compressive strength of the UHPC was 152.6 MPa, which already met the requirements for common UHPC. As for the HSC, its 56 day compressive strength was 119.5 MPa. Moreover, a residual compressive strength test was performed on cylindrical specimens exposed to heat from 300 to 500 °C and cooled to room temperature. The results are shown in Figure 6. In general, after experiencing a high temperature of 300–500 °C, the residual compressive strength of the HSC and UHPC only slightly decreased.

### 3.2. Measurement and Analysis of the Internal Temperature of the Beam Specimens

**Figure 7:**
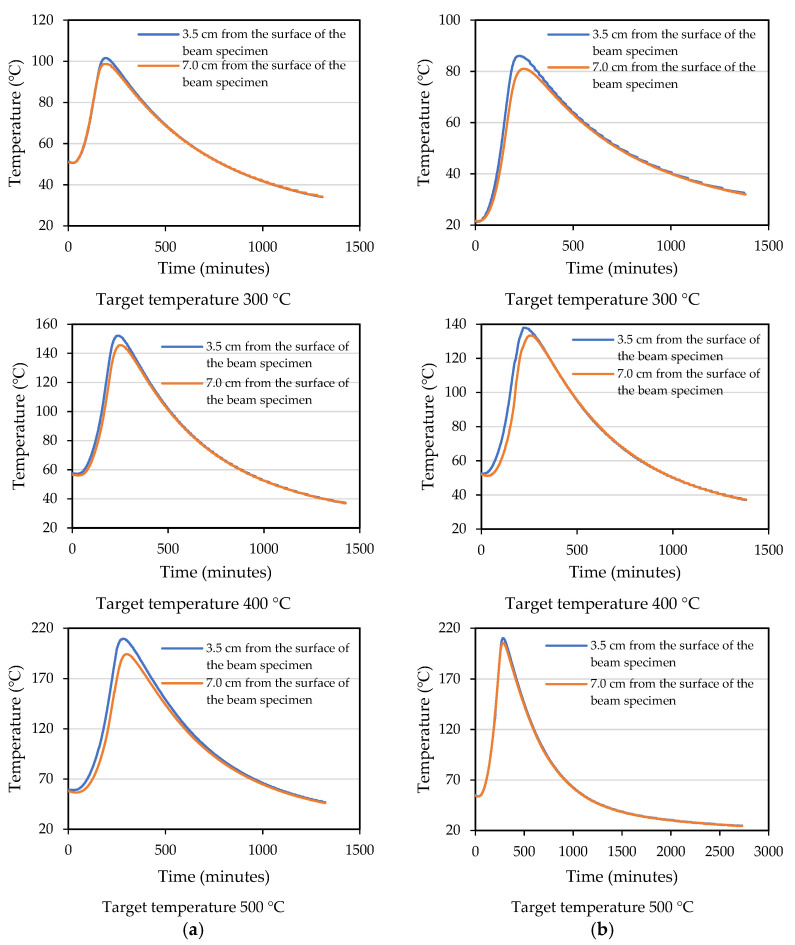
The relationship between the specimen internal temperature and time: (**a**) the experimental group and (**b**) the control group.

Under different target temperature conditions, the measured concrete temperature inside the beam specimen and the data showing the electric furnace temperature vs. time were plotted in Figure 7. It can be seen from Figure 7 that the concrete temperature measured in the mid-span section of the beam specimens of the HSC and UHPC was a function of the fire exposure time. In addition, the temperature of the concrete in the layer furthest from the fire-exposed bottom surface was lower than the temperature of the concrete in the layer closest to the fire-exposed surface. This trend is in line with our expectations due to the low thermal conductivity and high specific heat of concrete, which delays the transfer of temperature to the inner concrete layer. It can be seen from Figure 7 that when the three different target temperatures were reached, the maximum temperature inside the UHPC specimens was about 101.6, 152.1, and 209.4 °C. In other words, the temperature measured inside the concrete specimen was 58–66% lower than that of the furnace. Furthermore, the temperature difference between 3.5 and 7 cm from the surface of the concrete specimen was not significant. The same situation also occurred in the HSC.

**Figure 8:**
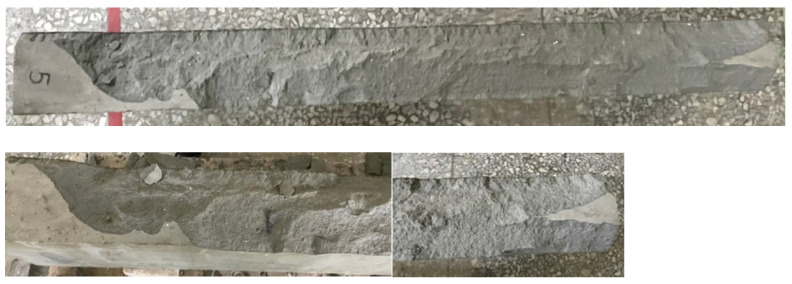
The spalled surface of the HSC beam specimen after being subjected to 500 °C.

**Figure 9:**
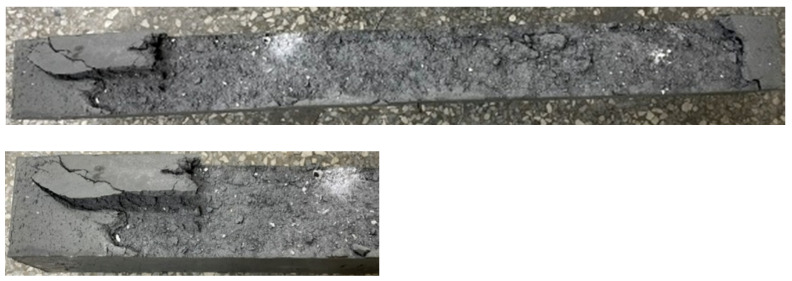
The spalled surface of the UHPC beam specimen after being subjected to 500 °C.

When the beam specimens were heated, the vapor pressure in their internal pores rose closer to the surface. Then, the pressure gradient not only expelled the moisture from the specimen, but also drove the moisture to the cooler areas inside. When the target temperature was set to 300 and 400 °C, the HSC and UHPC beam specimens did not spall. However, when the target temperature was set to 500 °C, the bottom surfaces of the HSC and UHPC beam specimens showed an explosive spalling phenomenon, as shown in Figure 8 and Figure 9, respectively. It could be seen from the thermocouples embedded in the specimens that the internal temperature of the HSC and UHPC beam specimens pre-dried in a furnace at 105 °C for 24 h rose to 54.5 °C, and there was no tendency to rise. From this, it can be seen that the moisture inside the test body did not completely evaporate, resulting in an explosive spalling of the corners of the bottom surface of the beam specimen at the target temperature of 500 °C. The UHPC beam specimen used polypropylene fibers to provide water vapor pressure relief channels. However, it is known from the literature [42] that the preferred amount of polypropylene fiber is about 0.1% to 0.15% (volume percentage). In this study, considering the workability and strength of concrete, the blended polypropylene fiber mass was only 0.06%, meaning that the effect of the water vapor pressure relief channel was relatively small.

It can be seen from the remaining fractions of concrete shown in Figure 8 that the bottom surface of the HSC specimen was simply the corner of the protective layer flake-off, and its range was relatively small. The spalling behavior of the bottom of the UHPC specimen was different. Due to the addition of steel fibers, when the steam pressure inside the beam was sufficient to produce explosive spalling the steel fibers inside the protective layer could provide tensile strength. However, in the case of excessive vapor pressure, the force and range of explosive spalling would also be much larger than that of the HSC specimen without steel fibers, as shown in Figure 9. In other words, the bottom protective layer of the UHPC specimen was completely collapsed. In contrast, the bottom protective layer of the HSC specimen dropped from the tiny corners. As the HSC specimen lacked the bridging effect of the steel fiber, it flaked off separately rather than at the same time. A subsequent three-point load test on the explosively spalling concrete beams was carried out to examine the degree of performance degradation after the fire spalling. In order to improve the excessive internal vapor pressure of the concrete beam at the target temperature of 500 °C, the second specimens of the HSC and UHPC beam were dried in a furnace at 130 °C for 48 h and the highest internal temperature recorded was 67.2 °C. After finishing the 48 h drying operation, the beam specimen was subjected to a high temperature of 500 °C, after which it was found that no more destructive spalling occurred. The study by Shorter and Harmathy [43] shows an important result; when a sample made of concrete that is known to spall is properly pre-dried to produce a thickness of about 50–65 mm, it will not spall at high temperatures. Therefore, they believe that it is reasonable to assume that for each type of concrete, a critical water content or water content distribution could be found, and no spall would occur below the critical water content or water content distribution. The results of this study are similar to this.

### 3.3. The Results of Flexural Test of Beams at Room Temperature

**Figure 10:**
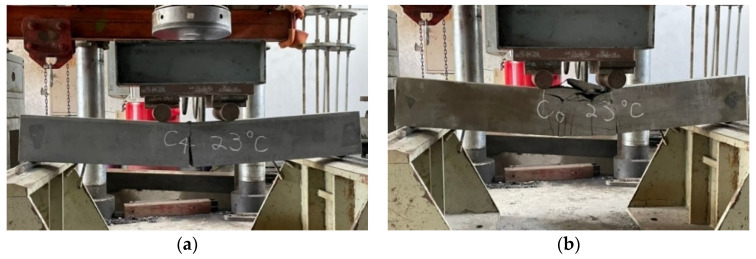
The tensile failure in the flexural test of beam at room temperature: (**a**) the experimental group and (**b**) the control group.

The configuration of the stirrups of the beam specimen was limited to the shear span. Its purpose was to prevent the specimen from being damaged by shear failure and cause the specimen to fail in tension so as to compare the flexural behavior of different concrete beams. As the HSC and UHPC beam members were designed to have a relatively low reinforcement ratio, the two groups of beam specimens were a tensile failure, as shown in Figure 10. When the load reached the flexural cracking load of the beam, vertical cracks began to appear in the pure bending moment area at the mid-span of the specimen, and the width was about 0.1–0.2 mm. As the load continued to increase, the steel fibers were pulled out one after another, the vertical cracks continued to extend upward, and their widths continued to increase to become obvious cracks. In addition, we could even hear the sound of fibers being pulled out, and we observed concrete fragments falling from the cracks. When the load exceeded the peak load, the load-bearing capacity of the specimen decreased significantly, while the crack width continued to increase and then penetrate the concrete on the compression side. The test results showed that when the surface of the concrete on the compression side in the pure moment zone flaked off, the residual load of the HSC beam could still be maintained for a period of time. The concrete strength of the UHPC was higher and steel fiber and polypropylene fiber were mixed. Therefore, the peak load and residual load of the UHPC specimen were significantly higher than those of the HSC. However, due to the configuration of the minimum reinforcement ratio, the failure of the UHPC beams was rapid, with a characteristic brittle cracking. The test results of the average peak load of the HSC and UHPC specimens at room temperature are shown in Table 8. It can be seen from Table 8 that, at room temperature, the 56-day-old average peak load of the UHPC beam was significantly higher than that of the HSC beam, with a gap of 27.4 kN. This result shows that the UHPC beam specimens made full use of the high tensile strength of the ultra-high-performance fiber-reinforced concrete, which benefited from the presence of steel fibers.

### 3.4. The Results of Flexural Test of Beams after Different Thermal Exposures

**Figure 11:**
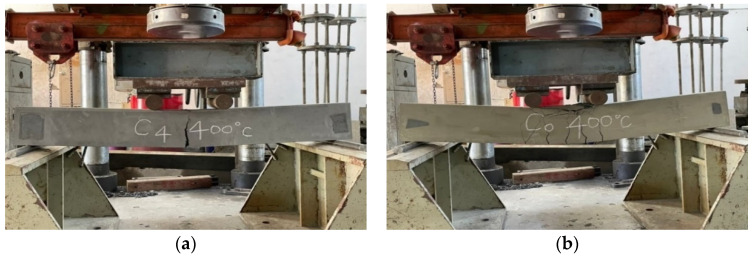
The tensile failure in the flexural test of beams after being subjected to 400 °C: (**a**) the experimental group and (**b**) the control group.

After the beam specimens were subjected to different thermal exposures and cooled, they were transported to the MTS universal testing machine in our large-scale structural laboratory for a three-point load test. After experiencing high temperatures, the beam specimens of the two groups of concrete still showed tensile failure. For example, Figure 11 shows the tensile failure of a beam in a flexural test after being subjected to 400 °C.

**Figure 12:**
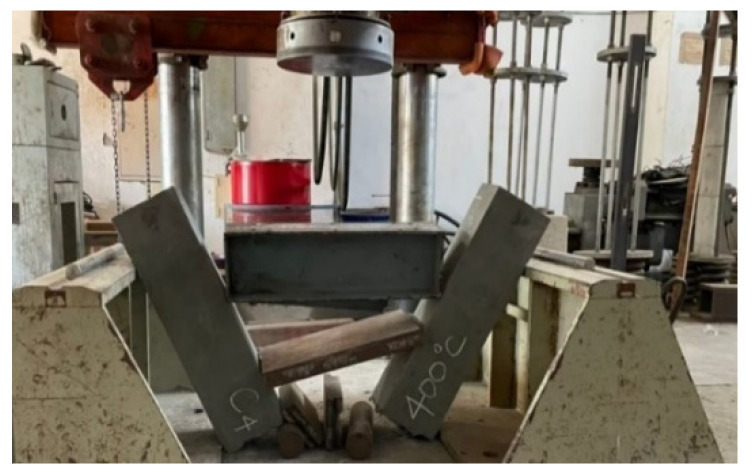
The fracture situation of the experimental group after being subjected to 400 °C.

**Figure 13:**
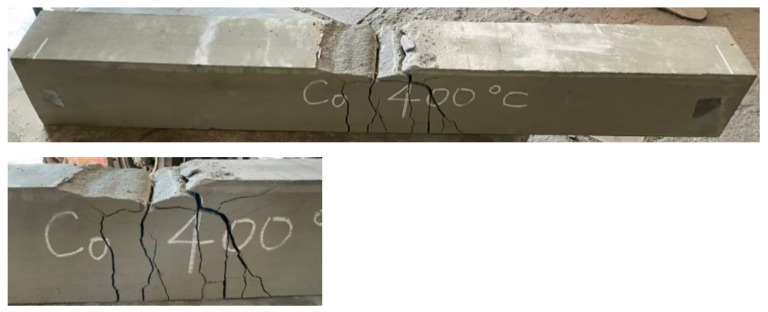
The distribution of the flexural cracks of the control group after being subjected to 400 °C.

The UHPC beams had a high compressive strength and were mixed with steel fiber and polypropylene fiber. There was only one vertical flexural crack in their pure bending moment zone, and there was no obvious crushing and flake-off of the concrete surface on the compression side. In addition, when the beam of the experimental group reached the ultimate load, the flexural cracks quickly passed through the pressure zone, causing the specimen to break into two halves, as shown in Figure 12. In contrast, the test results demonstrated that, due to the lower concrete compressive strength and the absence of steel fiber and polypropylene fiber in the HSC beam, there were many vertical flexural cracks in its pure bending moment zone. Furthermore, the concrete surface on the pressure side was obviously crushed and flaked off, as shown in Figure 13.

When the target temperature reached 300, 400, and 500 °C, it can be seen from Figure 7 that the maximum temperature of the internal concrete of the UHPC beams was about 101.6, 152.1, and 209.4 °C, respectively. From this point of view, when the target temperature was 300 or 400 °C, the internal temperature of the beam specimen was not enough to cause the attenuation of the concrete material, meaning the residual flexural load was not significantly reduced but rather increased. The data in Table 8 show that the average relative residual peak load ratio of the beam specimens after being subjected to 300 and 400 °C was as high as 0.94. In particular, as the target temperature reached 500 °C the average residual load still increased and the average residual peak load ratio after exposure to high temperatures was greater than one.

### 3.5. The Scanning Electron Microscopy Observation before and after Different Thermal Exposures

**Figure 14:**
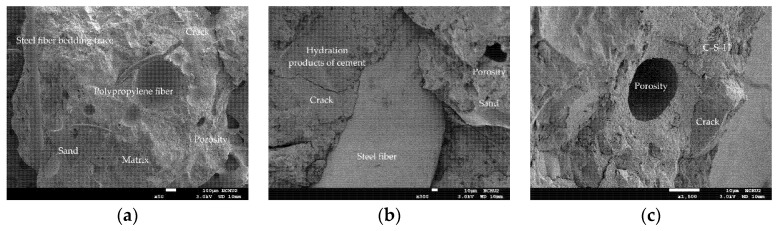
SEM observations of the UHPC sample at room temperature: (**a**) 50× magnification, (**b**) 300× magnification, and (**c**) 1500× magnification.

In order to further understand the changes in the microstructure of the HSC and UHPC specimens before and after high temperature exposure, a small amount of concrete fragment samples was collected from the surface of the beam specimen. The selected fragments were cleaned, dried, and gold-plated for scanning electron microscopy (SEM) observation at different magnifications. The micrographs of the UHPC sample at room temperature are shown in Figure 14. In the figure, steel fibers and polypropylene fibers distributed in the matrix can be observed. These fibers crisscrossed and formed a certain network structure which could effectively improve the tensile strength of the concrete. In addition, the higher magnification of Figure 14 reveals more or less connected crystal networks; the needles were hydration products of ettringite, the network flocs were hydrated calcium silicate (C-S-H) gels, and the black parts were the voids of the filler. Furthermore, it can be seen from Figure 14 that the fine aggregates were evenly distributed and well combined with the matrix, indicating that it had not undergone thermal degradation. In particular, the interfacial transition zone (ITZ) between the fine aggregate and the matrix was very dense with few air pores, and the thickness was very low. This is mainly due to the extra C-S-H produced by the pozzolanic reaction between ultra-fine silica powder and portlandite, which enabled them to fit between the grains of cement and the interface between paste and aggregate. Two main damage modes were identified in the ITZ. First, cracks of the matrix around the ITZ were observed. These cracks were caused by the stress transferred from the steel fiber to the matrix in the mechanical test. The cracks started in the matrix because the fracture strength of concrete was lower than that of the steel fiber. The other is that the steel fibers could also be observed to be debonded and pulled out of the matrix. At the position where the steel fibers were pulled out, no calcium hydroxide (Ca(OH)_2_) crystals were found. The reason is speculated to be that the interface area between the steel fiber and cement was filled with secondary hydration reaction products, meaning that the transition interface was not obvious [44].

**Figure 15:**
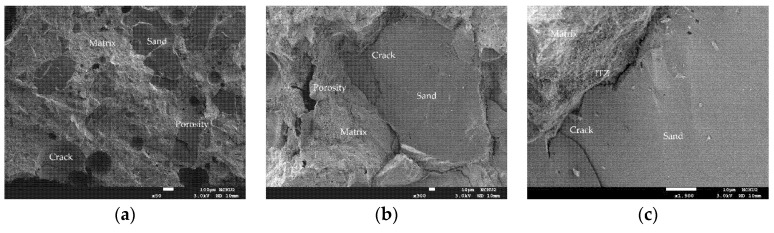
SEM observations of the HSC sample at room temperature: (**a**) 50× magnification, (**b**) 300× magnification, and (**c**) 1500× magnification.

As far as the room temperature condition is concerned, the micrographs of the HSC sample showed a typical concrete microstructure with dark gray aggregates and light gray unhydrated cement grains surrounded by cement paste, as shown in Figure 15. Compared with the UHPC sample, the HSC sample had more internal pores, which were characterized by the black cavities shown in the photomicrographs, indicating that their structure was relatively sparse. In addition, cracks and pores, which may have been caused during the mechanical test, appeared on the matrix in the sample.

**Figure 16:**
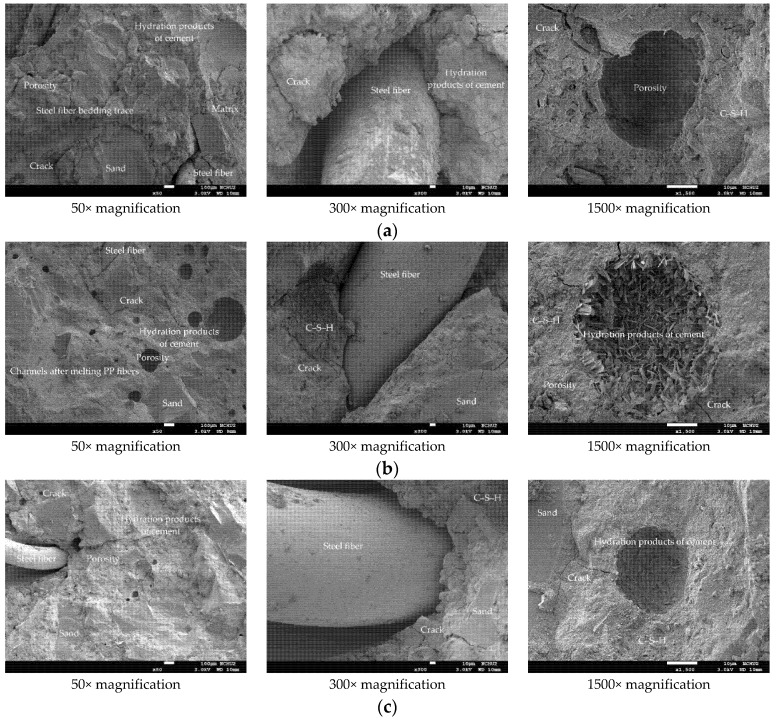
SEM observations of the UHPC sample after being subjected to high temperatures: (**a**) 300 °C, (**b**) 400 °C, and (**c**) 500 °C.

Generally speaking, when fiber-reinforced concrete suffers from fire, the thermal deformation of its components will cause the micro-cracks at the interface between cement paste and aggregate and between cement paste and fiber to open and expand [32]. In particular, the vapor pressure accumulated in the pores will cause internal stress, resulting in changes to the crystal structure and its volume [32]. Further, the dehydration phenomenon of calcium hydroxide may easily lead to the formation of shrinkage cracks. After exposure to high temperature, it is inevitable that the microstructure and performance of the UHPC matrix would also be affected and changed, as shown in Figure 16. In the sample, the spherical pores are filled with cement hydrates. The higher magnification of Figure 16 shows the presence of tobermorite on the inner wall of the sample’s pores. It can be seen from the figure that a large number of leaflet-like and needle-like tobermorite crystals interlace with each other to form a network, while some C-S-H gels act as a binder to connect them. Furthermore, lathlike crystals also interlock with each other to form a network. As the target temperature increases, the interface between the experimental group’s matrix and the steel fiber does not loosen significantly. However, SEM micrographs show that due to thermal stress, vapor pressure, and differential expansion between the matrix and the aggregate, the microcracks at the aggregate-matrix interface after exposure to high temperatures were wider than the cracks at room temperature. In addition to the aforementioned factors, the decomposition of Ca(OH)_2_, calcite, and C-S-H gels led to crack propagation and connection, which in turn increased porosity. In particular, in specimens that were severely damaged at high temperatures, the cracks could penetrate the matrix connecting the pores or bypass the pores. It can be seen from Figure 16 that as the temperature increased, the steel fiber did not melt. This is because the temperature measured inside the beam specimens was 58–66% lower than the temperature in the furnace. In addition, the cement paste wrapped around the steel fiber has cracks along the longitudinal direction of the steel fiber, but it still retains a certain degree of bond strength. Based on this, it is speculated that the steel fiber improved the tensile strength of the UHPC matrix to a certain extent and delayed the occurrence of explosive spalling at high temperatures, but it is not enough to completely alleviate it. In other words, steel fiber could reduce the risk of spalling caused by thermal stress, but the steel fiber bridge could not completely resist the increase in pore pressure. This is consistent with the findings of Du et al. [45] and Sciarretta et al. [46].

Figure 16 shows that under the action of high temperature, part of the ettringite and C-S-H gel in the sample was destroyed and dispersed. Moreover, acicular ettringite cracked due to thermal expansion and contraction. It can be seen from Figure 16a that at a target temperature of 300 °C, the polypropylene fibers in the matrix had already melted and could not be observed. When the target temperature was 400 or 500 °C, most of the internal bound water of the hydrate was lost. Taking C-S-H gel as an example, about 20% of bound water was lost [1,2]. At this time, from Figure 16b,c, the traces and holes left after the polypropylene fiber was melted could be clearly observed. The size of these holes was similar to that of the polypropylene fiber. This is mainly due to the melting of the polypropylene fibers at high temperatures, starting at about 150 °C and finishing at 176 °C [47,48]. Therefore, the polypropylene fibers could release the pore pressure to a certain extent to prevent the UHPC matrix from explosive spalling. In other words, polypropylene fibers melted at high temperatures and formed a pore network, which could not only effectively reduce the internal vapor pressure, but also maintained a certain residual strength. This is consistent with the research results shown in the literature [49,50].

**Figure 17:**
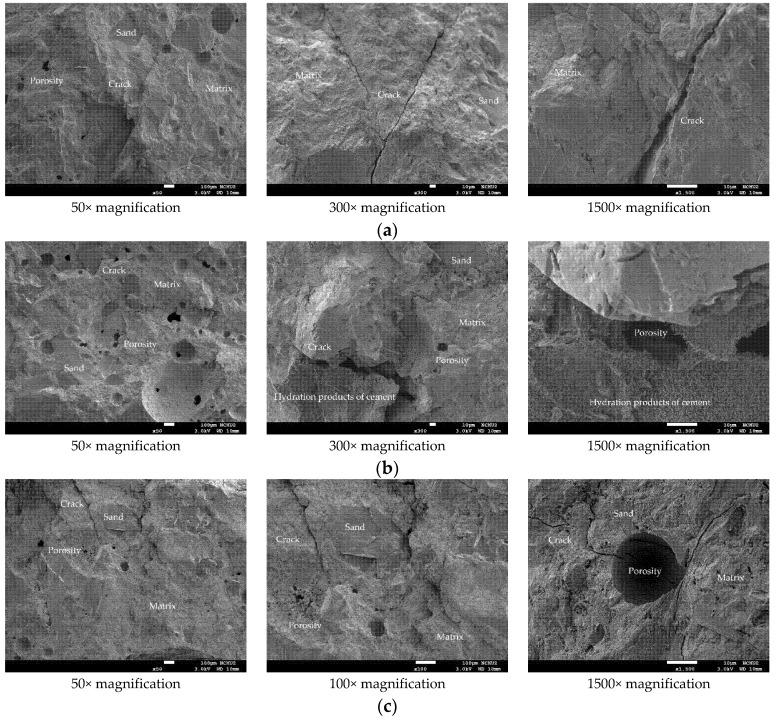
SEM observations of the HSC sample after being subjected to high temperatures: (**a**) 300 °C, (**b**) 400 °C, and (**c**) 500 °C.

After being exposed to different thermal exposures, the microstructure and performance of the HSC matrix also changed with the increase in temperature, as shown in Figure 17. Similarly, in the sample, it could be observed that the spherical pores were filled with cement hydrate. It can be seen from the sample shown in Figure 17 that when the target temperature reached 300 °C, the microstructure of the cement paste did not change significantly and only micro cracks appeared. When the target temperature reached 400 °C, there was basically no obvious internal damage except for slight burns on the surface that had been in contact with the fire. In addition, some fine cracks were observed at the interface between the aggregate and the cement matrix. At this time, the concrete did not yet show signs of damage. When the target temperature reached 500 °C, the sample was completely dehydrated, causing cracks to appear on the internal bonding surface. At this point, the internal stress of the concrete began to increase and microcracks began to appear in the sample. Therefore, the crack density here was much higher than the crack density produced by the previous temperature. In addition, the porosity also increased, mainly due to the shrinkage of the matrix related to the dehydration of C-S-H gels. Compared with the UHPC samples, the HSC samples showed more internal cracks after reaching high temperatures.

### 3.6. The Load–Deflection Relationship of Beam at Room Temperature

**Figure 18:**
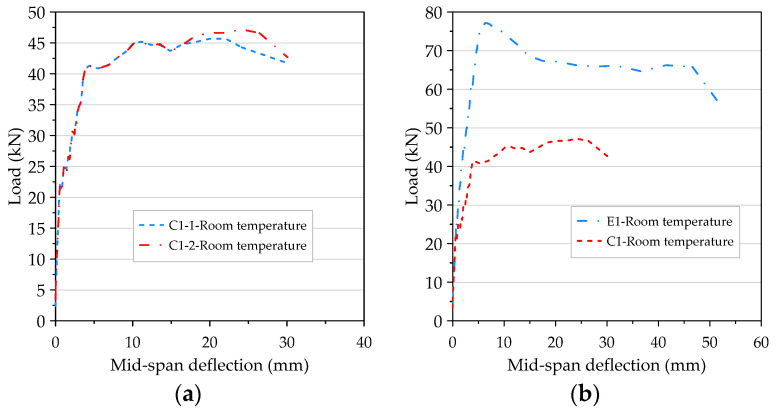
The load–deflection curve of beam: (**a**) the control group at room temperature and (**b**) comparison of the two groups at room temperature.

The load–deflection curves of the room temperature flexural testing of the HSC beams are shown in Figure 18a. Basically, the two beams showed several different response stages—namely, a linear elastic stage until the initial flexural cracking, a cracking stage after the initial flexural cracking and its progress, the beginning of the rebar yielding, a plastic deformation stage until the peak load was reached, then finally serious damage. It can clearly be seen from the figure that before the rebar reached the yield stress, the load–deflection curves of the two beams almost overlapped. Even after the rebar reached the yield stress, there was still a short section of the curve that showed the same development trend. Overall, when the bending moment caused by the load was less than the cracking moment of the beam section, the bending stress of the beam was less than the tensile strength of the concrete. At this time, the entire beam section could bear the stress. Below the neutral axis, the concrete and rebar shared the tensile stress, while above the neutral axis the concrete resisted the compressive stress. This was the elastic stage. When the load gradually increased, once the resulting bending moment reached the cracking moment of the beam section the flexural stress on the section was greater than the tensile strength of the concrete, resulting in flexural cracks in the pure bending moment zone. As the load continued to increase, the flexural cracks extended up to the neutral axis and the neutral axis also moved up with the cracks at this time. At the cracked section, the concrete could not bear the tensile stress, but the rebar resisted the tensile stress. At this stage, the beam was converted from an uncracked state to a cracked state. When the bending moment caused by the load reached the yield bending moment of the beam section, the rebar first reached the yield stress and then began to plastically deform. At the same time, the flexural cracks of the concrete widened rapidly and moved upward while the rate of increase of the load was reduced. When the strain value of the protective layer at the top of the beam in the pure bending moment zone was between 0.004 and 0.0055, the concrete of the protective layer was flaked off due to crushing and the load was slightly reduced. After that, the load continued to increase, and after reaching the peak the concrete on the compression side of the pure bending moment zone was broken and penetrated by flexural cracks, as shown in Figure 10b. Immediately, the load-bearing capacity of the beam dropped sharply and failed. A careful inspection of the failed beam revealed that the crack widths were not constant, which gradually decreased from the bottom to the top of the beam. This is mainly because the variability of crack width is not only related to the bending effect of the beam cross-section, but also closely related to the shear force of the beam. This result is consistent with the literature [51,52].

**Figure 19:**
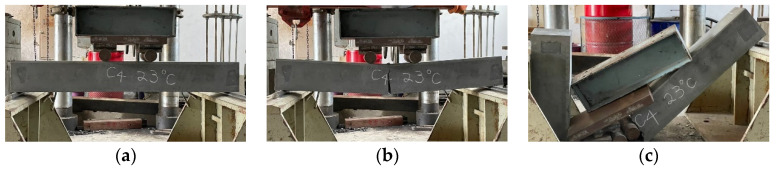
The failure process of the UHPC beam at room temperature: (**a**) elastic stage, (**b**) cracking stage, and (**c**) ultimate stage.

The load–deflection curve of the UHPC beam at room temperature is shown in Figure 18b. In the case of a low load, as long as the maximum tensile stress in the concrete is less than the cracking modulus, the entire beam section can effectively resist the stress. One side of the neutral axis was compressed and the other side was under tension. In addition, the rebar with the same amount of deformation as the adjacent concrete also resisted tensile stress. At this stage, the ascending branch of the load–deflection curve was quite linear. That is to say that before the first crack was formed, the stress in the concrete was small in magnitude and proportional to its strain, as shown in Figure 19a. When the load was further increased, the tensile strength of the concrete was reached soon and then a tensile crack occurred. The formed cracks quickly propagated upward to or close to the level of the neutral axis, and the neutral axis moved upward as the crack grew. At the cracks, the concrete could no longer withstand the tensile stress, but the tension was resisted by the steel bars, as shown in Figure 19a,b. When the load increased further, the stress and strain increased accordingly and were no longer proportional. In other words, a nonlinear relationship between stress and strain was created in the concrete stress–strain curve. The reinforcement ratio of the beam members of the experimental group was quite close to the minimum reinforcement ratio required by the ACI code. Therefore, when the ultimate bearing capacity of the beam was finally reached, the crack quickly penetrated the concrete on the pressure side, causing the beam to break into two sections, as shown in Figure 19a.

Compared with the HSC beams (excluding steel fibers and polypropylene fibers), the UHPC beams had a relatively higher stiffness after flexural cracking (as shown in Figure 18b). Under clearly identifiable peak loads, the UHPC beam did not fail in a brittle and catastrophic manner when the first crack was formed. Before the material showed obvious signs of damage, the load–deflection relationship of the beam became nonlinear, and microscopic examination of the specimen showed that some small cracks had formed. This is because the steel fiber used in this study has a higher specific strength. In addition, the maximum and ultimate load of the UHPC beams increased significantly and the deflection under a given load was small. These improvements were attributed to the high bond strength between the fiber and the matrix, which was achieved by the densification of the silica powder of the matrix and the low water–cement ratio [53]. In particular, the behavior of steel fiber as a crack bridge enabled the UHPC beam to bear the load appropriately after cracks appeared in the concrete [54].

### 3.7. The Load–Deflection Relationship of Beam after Different Thermal Exposures

**Figure 20:**
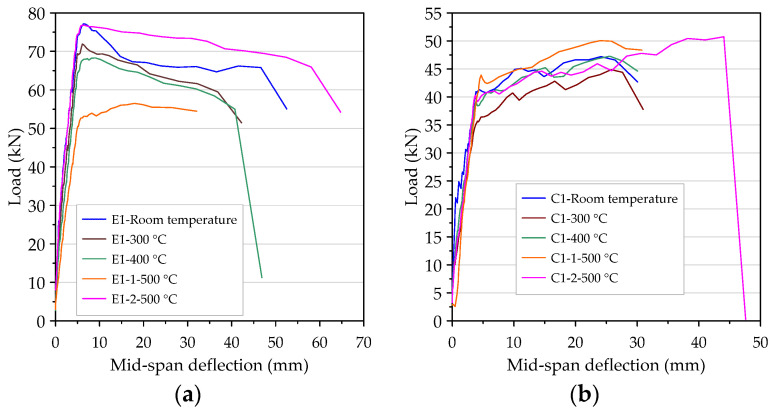
The load–deflection curve of the beam after being subjected to high temperatures: (**a**) the experimental group and (**b**) the control group.

As mentioned earlier, in order to improve the excessive internal vapor pressure of the concrete beam at the target temperature of 500 °C, the second specimens of the HSC and UHPC beam were pre-dried in a furnace at 130 °C for 48 h. After that, when the high-temperature application of 500 °C was performed again, it was found that the beam specimen did not destructively flake off. After exposure to different temperatures, the concrete performance was changed, but the flexural test results of the HSC and UHPC beams all showed tensile failures. Regarding the flexural test results of the beam specimens after exposure to different temperatures, the detailed load–deflection curves were compiled and are shown in Figure 20. It can be seen from Figure 20 that, according to the load, the load–deflection relationship curve of the beam specimen after experiencing high temperatures could be divided into the initial flexural cracking load, the rebar yielding load, the peak load, and the ultimate load. A similar pattern was reflected in the linear relationship between the load–deflection curve of the beam specimens before the flexural crack occurred. After the flexural crack occurred, it remained almost linear and its stiffness decreased slightly; however, when the longitudinal rebars yielded, its stiffness also decreased. It can be seen from Figure 20a that when the target fire temperature was between 300 and 400 °C, the stiffness of the UHPC beam (that is, the slope of the ascending branch of the load–deflection curve) was only attenuated by a small amount, compared with room temperature conditions. However, when the target temperature was 500 °C, the UHPC specimen E1-1-500 °C was dried in a furnace at a lower temperature of 105 °C for only 24 h, and the slope of the rising branch of its load–deflection curve showed a significant attenuation. In contrast, the UHPC specimen E1-2-500 °C was dried in a furnace at a higher temperature of 130 °C for 48 h, and its stiffness only slightly decreased. Overall, the initial cracking load and rebar yield load of the UHPC specimens did not change significantly after high temperatures, but the peak load and ultimate load were significantly attenuated, as shown in Figure 20a. However, the UHPC specimen (E1-2-500 °C) that had been pre-dried in a furnace at a temperature of 130 °C for 48 h showed only a slight decrease in the peak load and a significant increase in the ultimate load. In addition, after reaching the peak load, the rate of load reduction was significantly lower than that at room temperature. This result shows that reducing the moisture content of the UHPC beams is an effective way to improve its spalling resistance, but can also increase its residual flexural load. Similarly, the peak load and ultimate load of the HSC specimens (C1-2-500 °C) that had been pre-dried in a furnace at 130 °C for 48 h were significantly improved, as shown in Figure 20b.

**Figure 21:**
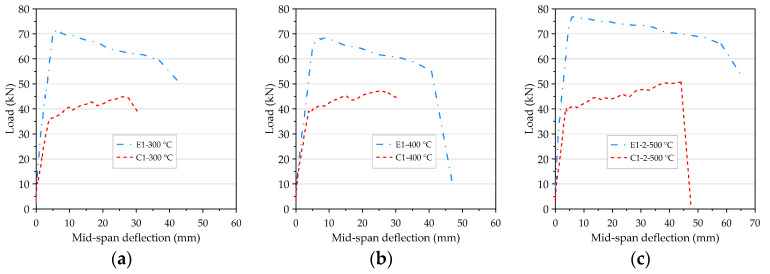
The comparison of load–deflection curves of two groups of beams after being subjected to high temperatures: (**a**) 300 °C, (**b**) 400 °C, and (**c**) 500 °C.

On the other hand, after different target temperatures, the initial cracking load, steel yield load, peak load, and ultimate load of the UHPC were significantly higher than those of the HSC, as shown in Figure 21. In addition, compared with the HSC specimens, the stiffness of the UHPC specimens in the post-cracking stage was relatively larger and the deflection under a given load was smaller.

### 3.8. The Ductility of the Beam Flexural Test after Different Thermal Exposures

Ductility characterizes the ability of reinforced concrete components to absorb energy and undergo significant inelastic deformation and can be measured according to the curvature, rotation, or deflection of the component. Exposure to high temperatures has a significant impact on the mechanical properties of UHPC [55]. In particular, high temperatures cause UHPC to exhibit brittleness. Therefore, it is necessary to further explore the influence of high temperatures on the ductility of UHPC beams. In order to simplify the calculation, the deflection ductility index [56] is often used. The expressions shown below are commonly used as ductility indices [57]:

(1) $$\mu_{p}=\frac{\Delta_{p}}{\Delta_{y}},$$

(2) $$\mu_{u}=\frac{\Delta_{u}}{\Delta_{y}}.$$

Among these, $\Delta_{p}$ is the mid-point deflection of the beam span under the peak load, $\Delta_{y}$ is the mid-point deflection of the beam span when the longitudinal reinforcement is yielded, and $\Delta_{u}$ is the mid-point deflection of the beam span under the ultimate load or the deflection corresponding to the load at 80–85% of the peak load from the descending branch of the load–deflection curve [58,59]. Qi et al. [60] proposed a new ductility index that divides the ultimate deflection by the flexural cracking deflection ($\Delta_{cr}$) to characterize the ductility of a component after cracking:

(3) $$\mu_{cr}=\frac{\Delta_{u}}{\Delta_{cr}}.$$

**Figure 22:**
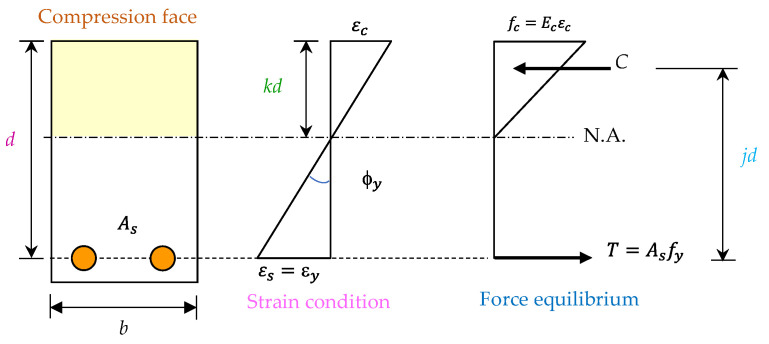
The stress and strain profiles of a typical reinforced concrete beam at the yielding state.

This study conducted a theoretical analysis on the ductility of the prepared beam specimens. When the longitudinal reinforcement of the beam yields, it reaches the yielding state. It can be considered that the prepared single-reinforced beam is basically in an elastic state. At this time, the stress and strain distribution of the beam in the yielding state is shown in Figure 22. Therefore, the yield curvature of the critical section of the beam ($\phi_{y}$) can be expressed as follows [61]:

(4) $$\phi_{y}=\frac{f_{y}}{E_{s}d\left(1-k\right)}.$$

where $f_{y}$ is the yield stress of steel rebar, $E_{s}$ is the modulus of elasticity of steel rebar, *d* is the effective depth of beam section, and *k* is the ratio of neutral axis depth to effective depth.

**Figure 23:**
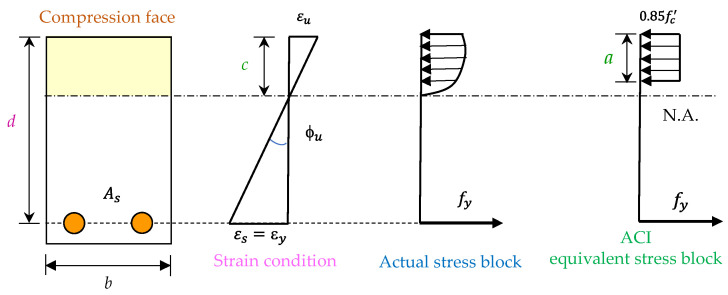
The stress and strain profiles of a typical reinforced concrete beam at the ultimate state.

The stress and strain distribution of the beam in the ultimate state is shown in Figure 23. The ultimate curvature ($\phi_{u}$) of the critical section of the beam can be expressed as follows [61]:

(5) $$\phi_{u}=\frac{\varepsilon_{u}}{c}=\frac{\beta_{1}\varepsilon_{u}}{a}.$$

where $\varepsilon_{u}$ is the ultimate strain of concrete, *c* is the depth of the neutral axis of the beam section in the ultimate state, $\beta_{1}$ is the ratio of depth of equivalent rectangular stress block to depth of the neutral axis, and *a* is the depth of equivalent rectangular stress block of the beam section.

**Figure 24:**
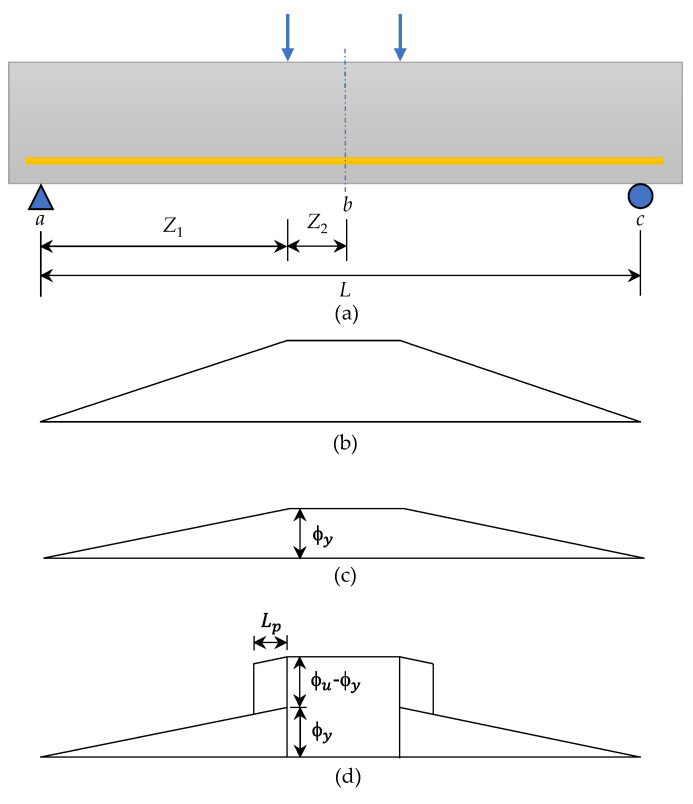
Schematic diagram of the curvature area theorem for obtaining a typical beam deflection: (**a**) load diagram, (**b**) bending moment diagram, (**c**) idealized curvature at the yielding stage, and (**d**) idealized curvature at the ultimate stage.

Consider a simply supported beam that bears two concentrated loads at the center of the span. Its bending moment diagram and curvature diagram are shown in Figure 24.

In reality, some cracks will be formed under the combined action of flexural stress and shear stress in the shear span of the beam [51,52]. Once a crack is formed, the bending stiffness of the beam will change along its axial direction. Due to the tension stiffening effect [62,63], the concrete between the cracks can withstand some tension. Therefore, the curvature fluctuates along the length of the beam. That is, each curvature peak corresponds to a crack position. In order to simplify the complexity of the calculation, the actual curvature distribution in the ultimate load stage can be idealized into elastic and inelastic (plastic) regions. According to the idealized curvature diagrams in Figure 24c, the relative yielding deflection ($\Delta_{yab}$) of the two points *a* and *b* on the beam can be determined by integrating the yielding curvature ($\phi_{y}$) of the cross-section along the length of the beam, as follows [61,64,65,66]:

(6) $$\Delta_{yab}=\phi_{y}\frac{Z_{1}^{2}}{3}+\phi_{y}Z_{2}\left(Z_{1}+\frac{Z_{2}}{2}\right).$$

where $Z_{1}$ is the distance from the supporting end to the concentrated load, and $Z_{2}$ is the distance from the critical section of the beam to the concentrated load. At the ultimate load stage, the actual distribution of curvature of a typical reinforced concrete beam can be idealized into elastic and plastic regions. According to the second moment area theorem [64], using the idealized curvature diagram in Figure 24d, the relative ultimate deflection ($\Delta_{uab}$) of the two points *a* and *b* on the beam can be determined as follows [61,64,65,66]:

(7) $$\Delta_{uab}=\phi_{y}\frac{Z_{1}^{2}}{3}+\left(\phi_{u}-\phi_{y}\right)L_{p}\left(Z_{1}-\frac{L_{p}}{2}\right)+\phi_{u}Z_{2}\left(Z_{1}+\frac{Z_{2}}{2}\right).$$

where $L_{p}$ is the equivalent length of the plastic zone. According to Mattock’s suggestion, the value of $L_{p}$ can be calculated by the following formula [67].

(8) $$L_{p}=0.5d+0.05Z.$$

where *Z* is the distance from the maximum bending section to the inflection point.

In this study, different ductility indices were calculated based on the load–deflection curve of the beam specimens. When the beam specimen was subjected to the flexural test, we closely monitored the development of the crack in the specimen to determine the position of the flexural crack that reached the cracking moment and the corresponding cracking deflection. In addition, $\Delta_{cr}$, $\Delta_{y}$, $\Delta_{p}$, and $\Delta_{u}$ could be obtained from the load–deflection relationship curve of the beam specimens, then $\mu_{cr}$, $\mu_{p}$, and $\mu_{u}$ could be calculated, as shown in Table 9. In addition, using Equations (6) and (7), the theoretical deflection ductility factor of the beam members at room temperature can be calculated, as shown in Table 9.

Generally, a larger ductility index indicates that the structural member can withstand greater deflection before failure. It can be seen from Table 9 that the average values of $\mu_{cr}$ of the HSC and UHPC beam specimens at room temperature were 36.66 and 34.35, respectively; meanwhile, the average values of $\mu_{cr}$ of the beams of the two groups after exposure to high temperatures were between 17.08 and 22.40 and 17.83 and 38.65, respectively. This result shows that the difference between the two was not significant. However, the average value of $\mu_{p}$ of the UHPC beams was less than that of the HSC beams regardless of whether it was at room temperature or after exposure to high temperatures. This was due to the relatively large stiffness of the UHPC beams, which made the $\Delta_{p}$ value smaller. On the other hand, structural members with $\mu_{u}$ between 3 and 5 are considered to have a sufficient ductility and can be considered for structural members with large displacements [68,69]. It can be seen from Table 9 that the average value of $\mu_{u}$ of the UHPC beams at room temperature was 12.43, while the average value of $\mu_{u}$ of the HSC beams at room temperature was 7.83. At room temperature, the average value of $\mu_{u}$ of the UHPC beams was 1.59 times the average value of $\mu_{u}$ of the HSC beams, which showed that the ductility of the UHPC beams was better.

After different thermal exposures, the average value of $\mu_{u}$ of the UHPC beams was between 8.32 and 16.42, while the average value of $\mu_{u}$ of the HSC beams was between 7.22 and 8.82. After exposure to 300, 400, and 500 °C, the average values of $\mu_{u}$ of the UHPC beams were 1.92, 1.15, and 1.25 times the average values of $\mu_{u}$ of the HSC beams, respectively. This result shows that the ductility of the UHPC beams was still better than that of the HSC beams after exposure to high temperatures. Overall, when the UHPC beams subjected to different levels of thermal exposure were subjected to larger flexural loads, the matrix wrapped around the steel fibers had cracks along the longitudinal direction of the steel fibers. However, it still maintained a good bonding state until the steel fiber tore under tension instead of debonding. In the process of failure, the deformation produced by the steel fiber dissipated part of the energy, which further increased the ductility of the UHPC beams. It can be seen from Table 9 that the values of $\Delta_{y}$ and $\Delta_{u}$ of the HSC beam at room temperature were 3.84 and 30.06 mm, respectively, while the theoretical analysis of the values of $\Delta_{y}$ and $\Delta_{u}$ were 3.17 and 46.47 mm, respectively. In addition, the values of $\Delta_{y}$ and $\Delta_{u}$ of the UHPC beam at room temperature were 2.57 and 31.95 mm, respectively, while the theoretical analysis of the values of $\Delta_{y}$ and $\Delta_{u}$ were 3.17 and 59.13 mm, respectively. These results show that there is a certain difference between the experimental results and the theoretical analysis results. This is because the judgment of yield deflection by visual method is more subjective, which makes it impossible to obtain accurate results. Similarly, the theoretical analysis also has errors as some assumptions simplify the complexity of the calculation.

## 4. Conclusions

According to the flexural behavior of the UHPC beams after being exposed to different levels of thermal exposure, the following conclusions can be drawn:

- After proper pre-drying treatment, the UHPC and HSC beam specimens did not spall even when exposed to a high temperature of 500 °C.
- SEM observation confirmed that the polypropylene fiber melted at high temperature, which increased the permeability of the UHPC matrix and released water vapor.
- At room temperature and after being subjected to different thermal exposures, compared with the HSC specimens, the stiffness of the UHPC specimens in the post-cracking stage was relatively larger and the deflection under a given load was smaller.
- The average relative residual peak load ratios of the UHPC beam specimens after being subjected to 300 and 400 °C were both as high as 0.94. In particular, as the target temperature reached 500 °C, the average residual load still increased, and the average residual peak load ratio was greater than one.
- The ductility of the UHPC specimens was better than that of the HSC specimens regardless of whether it was at room temperature or after exposure to high temperatures.
- Polypropylene and steel fibers can release pore pressure to a certain extent to prevent the UHPC matrix from spalling. Therefore, the UHPC beams incorporating hybrid polypropylene and steel fibers showed improved flexural performance after being subjected to different levels of thermal exposure. In addition, reducing the moisture content of the UHPC beams is an effective way to improve its spalling resistance.

The beam specimen in this study was configured as a single reinforced beam, which used the minimum reinforcement ratio to design the longitudinal tensile steel at the bottom of the specimen. Subsequent studies can be planned for different reinforcement ratios to further explore the flexural behavior of UHPC beams after high temperature. In addition, UHPC beams that are simultaneously subjected to load and high temperature can be planned to further explore the flexural behavior of UHPC beams.

## Figures and Tables

**Table 1:** The properties and compositions of the fine aggregates.

Type of Fine Aggregate:	Physical Properties		Chemical Composition		
	Specific Gravity (S.S.D.)	Water Absorption Rate (%) (S.S.D.)	Silicon Dioxide (%)	Iron Oxide (%)	Aluminum Oxide (%)
Type A	2.65	≈0	99.82	0.01	0.03
Type B	2.65	≈0	99.84	0.02	0.03

Notes: S.S.D., Saturated-Surface-Dry.

**Table 2:** The particle size distribution of the fine aggregates.

Sieve No. (ASTM E11-70)	Particle Size (μm)	Percentage Retained (%)	
		Type I	Type II
20	850	0.04	-
30	600	20.15	-
40	425	67.83	-
50	300	11.81	-
60	250	-	0.05
70	212	0.17	13.69
100	150	-	36.55
140	106	-	32.39
200	75	-	12.73
270	53	-	3.61

**Table 3:** The physical and mechanical properties of the fibers.

Type of Fiber	Length (mm)	Diameter (mm)	Density (g/cm^3^)	Elastic Modulus (GPa)	Tensile Strength (MPa)	Melting Point (°C)
Steel Fibers	13	0.2	7.8	200	2000	-
Polypropylene Fibers	12	0.05	0.9	-	300	165

**Table 4:** The physical and mechanical properties of the steel rebars.

Bar No.	Nominal Dia. (mm)	Nominal Cross Section Area (cm^2^)	Rib Distance (mm)	Rib Height (mm)	Yield Strength (N/mm^2^)	Ultimate Strength (N/mm^2^)
#3	9.53	0.713	8.3	0.7	334	464
#4	12.70	1.267	8.3	0.7	380	465

**Table 5:** The mix proportions of the concretes.

Designation	W/B	W	C	SF	SFP	SP	VA	PP	Steel Fiber	FA
	(kg/m^3^)									
C1	0.20	196	1005	0	0	26	1	0	0	1286
E1	0.20	186	756	179	21	25	1	0.5	78	1223

Notes: W/B, water–binder ratio; W, water; C, cement; SF, silica fume; SFP, ultra-fine silica powder; SP, superplasticizers; VA, viscous agent; PP, polypropylene fiber; FA, fine aggregate.

**Table 6:** The test methods of the fresh and hardened properties of the UHPC.

Property	Experiment Method
Unit weight	ASTM C138
Slump	ASTM C143
Slump flow	ASTM C1611
Compressive strength	ASTM C39
Flexural strength	ASTM C1609/C1609M-19a

**Table 7:** The test results of the fresh properties and compressive strength of concrete.

Designation	Slump (mm)	Slump Flow (mm)	Unit Weight (kg/m^3^)	56-Day Compressive Strength (MPa)
C1	262	690	2318	119.50
E1	257	540	2316	152.60

**Table 8:** The flexural load of the beams at room temperature and after thermal exposure.

Designation	Average Peak Load (kN)	Average Residual Peak Load (kN)			Average Relative Residual Peak Load Ratio		
	Room Temperature	Target Temperature			Target Temperature		
		300 °C	400 °C	500 °C	300 °C	400 °C	500 °C
C1	46.4	47.6	49.2	50.7	1.04	1.08	1.11
E1	73.8	75.3	69.3	76.8	1.02	0.94	1.04

**Table 9:** The average deflection ductility indices of beams.

Beam Designation	First Cracking Stage		Rebar Yield Stage		Peak Stage		Ultimate Stage		Deflection Ductility Indices			Theoretical Deflection Ductility Index		
	Load (kN)	$\Delta_{cr}\;(\mathrm{mm})$	Load (kN)	$\Delta_{y}\;(\mathrm{mm})$	Load (kN)	$\Delta_{p}\;(\mathrm{mm})$	Load (kN)	$\Delta_{u}\;(\mathrm{mm})$	$\mu_{cr}=\frac{\Delta_{u}}{\Delta_{cr}}$	$\mu_{p}=\frac{\Delta_{p}}{\Delta_{y}}$	$\mu_{u}=\frac{\Delta_{u}}{\Delta_{y}}$	$\Delta_{yab}$	$\Delta_{uab}$	$\frac{\Delta_{uab}}{\Delta_{yab}}$
C1-RT	21.07	0.82	40.88	3.84	46.44	22.06	42.19	30.06	36.66	5.74	7.83	3.17	46.47	14.66
C1-300 °C	23.28	1.93	38.32	3.86	47.55	26.39	42.95	32.96	17.08	6.84	8.54	-	-	-
C1-400 °C	21.23	1.38	42.07	3.66	49.14	21.64	47.89	26.44	19.16	5.91	7.22	-	-	-
C1-500 °C	19.80	1.67	42.09	4.24	50.39	34.02	49.55	37.41	22.40	8.02	8.82	-	-	-
E1-RT	25.93	0.93	45.47	2.57	73.79	7.01	61.09	31.95	34.35	2.73	12.43	3.17	59.13	18.65
E1-300 °C	26.00	1.10	45.33	2.59	75.12	6.88	47.33	42.52	38.65	2.66	16.42	-	-	-
E1-400 °C	25.84	1.53	45.77	3.28	69.23	8.82	61.87	27.28	17.83	2.69	8.32	-	-	-
E1-500 °C	26.03	2.56	45.46	4.50	66.62	13.39	54.32	49.49	19.33	2.98	11.00	-	-	-

## Data Availability

The data presented in this study are available on request from the corresponding author.
